# Data Fusion Algorithms for Multiple Inertial Measurement Units

**DOI:** 10.3390/s110706771

**Published:** 2011-06-29

**Authors:** Jared B. Bancroft, Gérard Lachapelle

**Affiliations:** Department of Geomatics Engineering, Schulich School of Engineering, University of Calgary, 2500 University Drive NW, Calgary, AB T2N 1N4, Canada; E-Mail: Gerard.Lachapelle@ucalgary.ca

**Keywords:** INS, sensor fusion, federated filter, pedestrian navigation

## Abstract

A single low cost inertial measurement unit (IMU) is often used in conjunction with GPS to increase the accuracy and improve the availability of the navigation solution for a pedestrian navigation system. This paper develops several fusion algorithms for using multiple IMUs to enhance performance. In particular, this research seeks to understand the benefits and detriments of each fusion method in the context of pedestrian navigation. Three fusion methods are proposed. First, all raw IMU measurements are mapped onto a common frame (*i.e.*, a virtual frame) and processed in a typical combined GPS-IMU Kalman filter. Second, a large stacked filter is constructed of several IMUs. This filter construction allows for relative information between the IMUs to be used as updates. Third, a federated filter is used to process each IMU as a local filter. The output of each local filter is shared with a master filter, which in turn, shares information back with the local filters. The construction of each filter is discussed and improvements are made to the virtual IMU (VIMU) architecture, which is the most commonly used architecture in the literature. Since accuracy and availability are the most important characteristics of a pedestrian navigation system, the analysis of each filter’s performance focuses on these two parameters. Data was collected in two environments, one where GPS signals are moderately attenuated and another where signals are severely attenuated. Accuracy is shown as a function of architecture and the number of IMUs used.

## 1. Introduction

As GPS markets continue to expand and new applications are found every day, any new application must abide by a key requirement, namely direct line-of-sight between the satellites and the receiver. So stringent is this requirement that the simple occlusion of satellites renders many navigation systems useless or highly degraded. As users travel in urban canyons, parkades, indoors or in high foliage areas, the ability for GPS to provide a navigation solution is compromised. Although High Sensitivity GPS (HSGPS) receivers can track weak signals through fading, this renders them susceptible to high noise and multipath errors [1]. Thus, researchers are examining other sensors to integrate with GPS.

Inertial measurement units (IMU) are a common complement to GPS, although it is technically more correct to state that GPS augments an inertial navigation system (INS). The advantage being that together the GPS and inertial sensors can provide a continuous navigation solution, where GPS alone cannot. As competitive consumer markets drive the price of mobile navigation devices lower, an increasingly common choice for IMUs is micro electro-mechanical systems (MEMS). Their size, cost, weight and low power consumption make them an attractive grade of IMU; however their in-run biases, scale factors and high noise require an effective integration scheme to mitigate these errors [2].

While existing INS research has involved one IMU, the purpose of this paper is to investigate the use of multiple IMUs in tandem with GPS. In particular, this paper will investigate various approaches to integrate multiple IMUs with several filter architectures and constraints that can be used to further improve the accuracy and availability of the navigation solution, with emphasis on pedestrian navigation.

The objectives of this paper, which is based on [3], are to:

1. Discuss the implementation and test results of the following techniques to utilize multiple IMUs and GPS observations for pedestrian navigation:
  - Virtual IMU observation fusion
  - Centralized filter design
  - Federated filter design
2. Assess fault detection capability on the IMU and GPS measurements, discussing any limitations.
3. Analyze and compare the performance of the different estimation architectures selected and the number of IMUs used.
4. Analyze the performance of each architecture in residential and indoor conditions.

### 1.1. Pedestrian Navigation

Potential pedestrian navigation users include: first responders (e.g., emergency search and rescue), cellular phone users (E911 and navigation), health and activity monitoring, recreational users (e.g., hikers, climbers, skiers), self-guided tourists, athletes and athletic trainers, consensual tracking (e.g., elderly, parolees, employees), navigation for the visually impaired and police/military forces.

A key to the success of many INS pedestrian navigation applications is the placement of the IMU on a foot (e.g., [4]) where the IMU experiences the repetitive and predictable motion of the human gait during walking. This allows for zero velocity updates while the foot in is contact with the ground, which plays a critical role in maintaining the long term accuracy of the system. Examples of this method can be found in [5–15].

This configuration reduces the necessity for magnetometers, although these can be used to aid with attitude determination as in [12]. The INS method also allows for direct analysis of sport and biomedical applications such as gait kinematics and posture analysis [16–19]. However, a disadvantage to this approach is the time varying lever arm between the GPS antenna and IMU(s). To date, this error has been ignored and thus the magnitude of the lever arm’s effect has not been quantified. Another limitation to the foot-mounted INS is the degraded accuracy over extended time periods. This result is common to all low cost INS setups and is primarily due to heading errors [5].

GPS and IMUs have been successfully integrated since the formal introduction of GPS. More recently, attention has been placed on integration with MEMS IMUs to reduce cost, but still provide robust navigation solutions. A natural progression is to use more IMU sensors and thus capitalize on the decreasing cost of MEMS sensors in order to improve overall accuracy. As such, researchers commonly fuse multiple IMU measurements in the raw observation (*i.e.*, specific force and angular velocity) domain, but have not pursued any other fusion methods. Thus, multi-IMU fusion can either occur in two categorical domains: the observation or estimation domain.

## 2. Raw IMU Observation Fusion

Numerous studies have taken an observation domain approach to redundant IMU (RIMU) integration whereby the observations of several IMUs are fused, generating a single virtual IMU measurement [20–29]. The term virtual IMU (VIMU) will be used herein to describe fusion architectures in the observation domain. RIMU is commonly used in the literature and can be confused with reduced IMU which has the same acronym.

In the development of VIMU theory, optimizing the configuration of the IMU sensor axes is an important consideration. Pejsa mathematically determined the optimal configuration for sensor axes; with sensors in a skewed formation rather than an orthogonal one (although the ideal 3-axis sensor is orthogonal) [30]. This optimal setup was named the Skew Redundant IMU (SRIMU). Further work derived the GDOP (Geometric Dilution of Precision) for a multi-sensor cluster to provide theoretical estimations, incorporating correct weighting schemes and providing fault detection through statistical misclosure testing [20,21].

**Figure 1.**
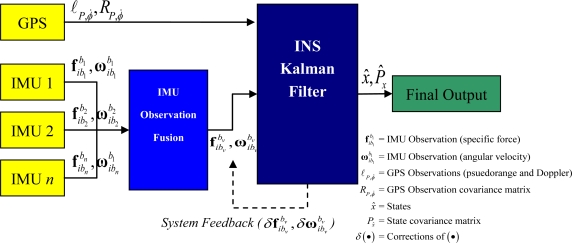
Virtual IMU Observation Fusion Architecture.

The prominent method of RIMU fusion fuses raw IMU observations using least squares estimation, mapping each IMU observation to a virtual IMU frame (which requires *a priori* knowledge of the transformation into the virtual fame). The methodology is described in [23,24,29]. However, this methodology is fundamentally flawed in that the IMU observations contain un-modeled errors prior to fusion and fault testing thus negating fundamental rules of input/output covariance estimation. Figure 1 shows the VIMU observation fusion and integration with GPS.

Often, the purpose of virtual IMU integration is not to improve the accuracy (although this is a desirable outcome), but rather to facilitate the detection and exclusion of faulty observations [20,22]. In many cases, such as aviation multi-IMU navigation systems, the purpose of adding additional IMUs to a navigation system is to facilitate IMU fault detection rather than improving accuracy. For pedestrian navigation applications, the opposite is true. Improving accuracy and availability are more important than high levels of reliability, although the latter also becomes important as soon as accuracy and availability requirements are met. This is most often the case because most pedestrian applications are not generally required to meet strict safety-of-life standards. Therefore, it will be shown herein that accuracy is improved through the use of a virtual IMU architecture. However, the validity and practicality of FDE may not be acceptable for low cost IMUs and their applications.

Another benefit of the virtual IMU scenario is a direct real time estimate of the VIMU process noise, as derived from each IMU [31]. This is beneficial when the IMUs have time variant process noise characteristics or filter tuning is not possible for each application or data set.

Averaging of IMUs’ observations is simple and the least computationally burdensome method of forming a VIMU, however because each IMU is located at a different point on the body, the IMUs measure different specific forces relative to the location of the VIMU origin. Consequently, the fusion must be performed in the same reference frame and the transformation of each gyro and accelerometer observation set into this frame must be performed. The transformation is assumed to be known *a priori* from pre-surveyed parameters, namely the vector between the IMUs and VIMU origin and the rotation from one IMU’s frame to the VIMU’s frame. From Kane and Levinson [32], the rigid body equations of the angular velocity from a VIMU are:

(1) $${\text{ω}}_{{\text{ib}}_{\text{n}}}^{{\text{b}}_{\text{n}}}={\text{R}}_{\text{v}}^{\text{n}}{\text{ω}}_{{\text{ib}}_{\text{v}}}^{{\text{b}}_{\text{v}}}$$

where ${\text{ω}}_{{\text{ib}}_{\text{n}}}^{{\text{b}}_{\text{n}}}$ is the angular velocity of the n^th^ IMU in its body frame, ${\text{R}}_{\text{v}}^{\text{n}}$ is the rotation matrix from the VIMU body frame to the body frame of the n^th^ IMU (known a priori) and ${\text{ω}}_{{\text{ib}}_{\text{v}}}^{{\text{b}}_{\text{v}}}$ is the angular velocity of the VIMU in the VIMU body frame.

The specific force, as derived from a VIMU relative to a rigidly attached body, is given as [32]:

(2) $${\text{f}}_{{\text{ib}}_{\text{n}}}^{{\text{b}}_{\text{n}}}={\text{R}}_{\text{v}}^{\text{n}}{\text{f}}_{\text{ib}}^{{\text{b}}_{\text{v}}}+{\text{R}}_{\text{v}}^{\text{n}}\left({\text{α}}_{\text{ib}}^{{\text{b}}_{\text{v}}}\times {\text{r}}_{\text{nv}}^{{\text{b}}_{\text{v}}}\right)+{\text{R}}_{\text{v}}^{\text{n}}\left({\text{ω}}_{{\text{ib}}_{\text{v}}}^{{\text{b}}_{\text{v}}}\times \left({\text{ω}}_{{\text{ib}}_{\text{v}}}^{{\text{b}}_{\text{v}}}\times {\text{r}}_{\text{nv}}^{{\text{b}}_{\text{v}}}\right)\right)$$

where ${\text{f}}_{{\text{ib}}_{\text{n}}}^{{\text{b}}_{\text{n}}}$ is the specific force vector of the n^th^ IMU, ${\text{f}}_{\text{ib}}^{{\text{b}}_{\text{v}}}$ is the specific force vector of the virtual IMU, ${\text{α}}_{\text{ib}}^{{\text{b}}_{\text{v}}}$ is the angular acceleration of the VIMU, and ${\text{r}}_{\text{nv}}^{{\text{b}}_{\text{v}}}$ is the lever arm vector between the n^th^ IMU and VIMU origins within the VIMU body frame.

To the authors’ knowledge, the second and third term on the right hand side of Equation (2) have been neglected in previous VIMU systems proposed in the literature. This adjustment to the mapping equation presents an important improvement in accuracy. Equation (2) uses the angular acceleration of the virtual frame, which may or may not be output by an IMU. In the event that the angular acceleration is not output by the IMU (as is the case herein), the angular acceleration must be estimated as an additional component of the VIMU fusion procedure in order to correctly determine the specific force. A nine-state estimation model is now described for the estimation of angular accelerations, in addition to the angular velocities and specific forces.

### 2.1. Nine-Parameter VIMU Least-Squares Estimator

In the VIMU least-squares model, the unknown parameters are the angular velocity, angular acceleration and specific force vectors of the VIMU. As a result of the cross products within Equation (2), the 9 state model is non-linear and therefore the system must be linearized. The linearized observation equation is:

(3) $$\left[\begin{array}{c} \partial {\text{ω}}_{\text{n}}\\ \partial {\text{f}}_{\text{n}} \end{array}\right]=\left[\begin{array}{ccc} {\text{R}}_{\text{v}}^{\text{n}} & 0 & 0\\ -{\text{R}}_{\text{v}}^{\text{n}}\text{A} & {\text{R}}_{\text{v}}^{\text{n}} & -{\text{R}}_{\text{v}}^{\text{n}}\left[\text{r}\times \right] \end{array}\right]\;\left[\begin{array}{c} \partial {\text{ω}}_{\text{v}}\\ \partial {\text{f}}_{\text{v}}\\ \partial {\text{α}}_{\text{v}} \end{array}\right],\;\;\forall \text{n}\in \left\{1,\ldots ,\text{N}\right\}$$

where A = [**ω**_v_ ×][**r** ×] + [(**ω**_v_ × **r**) ×] and N is the number of IMUs. The form [a ×] refers to the skew symmetric matrix of the vector a, which has the form a_3×1_ × b_3×1_ = [a ×]_3×3_b [33].

The nine parameter least-squares estimation operates in a standard fashion. It uses all gyro and accelerometer measurements as observations and provides an estimation of the virtual IMU accelerometer and gyro measurements. If five IMUs are used, then the system has 30 observations and operates at the same frequency as the incoming observations. Measurements were weighted equally because the IMUs are all the same brand and model, although this is not a requirement.

This nine-parameter least-squares model has a unique characteristic when two IMUs are used. When using two IMUs, the design matrix will only ever have a maximum rank of eight, indicating that only eight of the nine parameters are actually solvable. Conceptually, the linear dependency arises due to the fact that any angular acceleration about the vector between the two IMUs (*i.e*., the angular velocity vector and the vector between the IMUs is parallel) will result in zero acceleration. Therefore, all three-axis components of the angular acceleration cannot be estimated. As additional IMUs are added the angular acceleration between the two IMUs is observable from other non-parallel angular velocities.

### 2.2. Nine-Parameter VIMU Kalman Filter

The angular acceleration is the time derivative of the angular velocity and therefore a differential equation exists that relates these states. This forms the basis of a VIMU Kalman filter. A VIMU Kalman filter further reduces noise and can enhance navigation performance. The differential equations of the nine states are as follows:

(4) $${\left({\dot{\text{ω}}}_{\text{v}}\right)}_{\text{ib}}^{\text{b}}={\left({\text{α}}_{\text{v}}\right)}_{\text{ib}}^{\text{b}}$$

(5) $${\dot{\text{f}}}_{\text{v}}={\text{η}}_{\text{f}}$$

(6) $${\dot{\text{α}}}_{\text{v}}={\text{η}}_{\alpha }$$

where **η_f_** is the process noise of the uncertainty in the time derivative of the specific force vector and **η_α_** is the process noise of the uncertainty in the time derivative of the angular acceleration.

Determining the optimal values for **η_f_** and **η_α_** is challenging, given the time variant dynamics of the foot throughout the gait cycle. To resolve this issue, an adaptive Kalman filter is used to determine the process noise in real time. A 0.5 s window is used to determine the process noise. The observation variance is not derived from the adaptive filter, but is held constant to a pre-determined value.

The filter predicts and updates at the same frequency as the incoming measurements (*i.e.*, 100 Hz) which makes this version of the VIMU fusion the most computationally expensive. Updates are performed in an “epoch” mode (all measurements at a given epoch), although it is conceivable to process them sequentially for optimal processing speed.

The VIMU filter must operate with IMUs which are time synchronized. The adaptive Kalman filter could still function if the IMUs are synchronized but output observations at different data rates or if the observations had different time stamps. The required time synchronization is related to the angular dynamics, specifically the angular acceleration, and will incorrectly determine the specific force at the VIMU location.

### 2.3. Fault Detection and Exclusion (FDE) of VIMU Errors

This section will demonstrate that FDE is not always a viable option for MEMS IMUs with large biases, scale factors and acceleration-based gyroscope errors, in particular when IMUs experience significant accelerations and angular velocities. Fault detection works on the premise that the misclosure or innovation sequence is zero mean. As the biases and scale factors of each IMU have not been estimated, and therefore not removed from the observations, the observation model is not zero mean and therefore FDE effectiveness is compromised.

**Figure 2.**
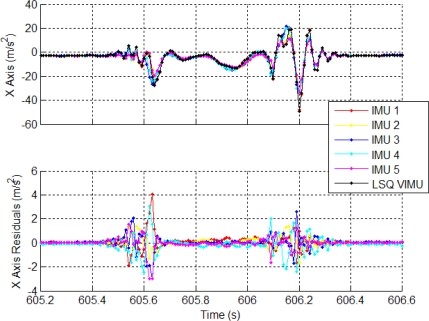
Specific Force Residuals from a Virtual IMU Computed from Least-Squares Estimation.

Residuals computed from a nine-state least-squares estimation of each sensor axis are shown in Figure 2. The period shows a complete gait cycle when all the IMUs are rigidly mounted on the foot. The residuals are shown with the raw IMU measurements of each sensor in the VIMU frame. The residuals for the accelerometer have a peak magnitude of about 4 m/s^2^, which corresponds to the highest acceleration within the gait cycle. Large gyro residuals of nearly 20 °/s are also observed and also correspond to high dynamics. During the stance phase of the gait, the residuals are much smaller, often in the range of the biases. Therefore, the magnitude of the residuals is clearly correlated to high dynamics.

Because the magnitude of the residuals is a function of dynamics rather than sensor errors, the input covariance matrix must accommodate these large variations, otherwise faults will be detected during every gait cycle (or whenever the IMU experiences high dynamics). With a VIMU architecture, each IMUs sensor error cannot be modeled individually. Thus, if FDE was to be performed, the input covariance matrix would not be a function of sensor noise, but rather have to contain an increased amount of error to account for uncorrected sensor errors. Therefore it is a recommendation herein that FDE *not* be performed on MEMS-based VIMU fusion.

## 3. Centralized Filter Fusion

This multi-IMU approach uses a centralized filter that is composed of several individual block filters (e.g., [5,7,24,34]). The technique allows for the inclusion of relative geometry constraints, such as relative position, velocity and attitude between IMUs. The use of these constraints represent an advantage over the VIMU estimation techniques since VIMU architectures fail to utilize this valuable information.

The centralized filter proposed in this paper is referred to as a stacked filter, consisting of several individual INS filters. In this manner several “block” filters (*i.e.*, Single INS filters) are contained within one centralized filter, ultimately operating as one.

The stacked filter contains parameters for position, velocity, attitude, accelerometer and gyro biases and accelerometer and gyro scale factors for each IMU. If five IMUs are used, then there are five 21-states filters contained within one centralized 105 state filter. Each block filter can be updated at the same time or individually, but the entire filter prediction cycle must be synchronized (to avoid different block times, within the stacked filter). An advantageous characteristic of the stacked filter (and federated filters) is that each block filter could contain additional or different IMU error states, thus facilitating varying types and qualities of IMUs and error state models, which the VIMU architecture does not. Since the IMUs are all the same brand and model, the block filters are identical with slightly varied input process noise parameters for each IMU. The block form of the stacked filter is:

(7) $$\left[\begin{array}{c} \partial {\text{x}}_{k+1}^{1}\\ \partial {\text{x}}_{k+1}^{2}\\ \vdots \\ \partial {\text{x}}_{k+1}^{n} \end{array}\right]=\left[\begin{array}{cccc} \Phi_{k,k+1}^{1} & 0 & 0 & 0\\ 0 & \Phi_{k,k+1}^{2} & 0 & 0\\ 0 & 0 & \ddots & 0\\ 0 & 0 & 0 & \Phi_{k,k+1}^{n} \end{array}\right]\;\left[\begin{array}{c} \partial {\text{x}}_{k}^{1}\\ \partial {\text{x}}_{k}^{2}\\ \vdots \\ \partial {\text{x}}_{k}^{n} \end{array}\right]+\left[\begin{array}{c} {\text{w}}_{k}^{1}\\ {\text{w}}_{k}^{2}\\ \vdots \\ {\text{w}}_{k}^{n} \end{array}\right]$$

(8) $$\left[\begin{array}{c} \partial {\text{z}}_{k+1}^{1}\\ \partial {\text{z}}_{k+1}^{2}\\ \vdots \\ \partial {\text{z}}_{k+1}^{n} \end{array}\right]=\left[\begin{array}{cccc} H_{k+1}^{1} & 0 & 0 & 0\\ 0 & H_{k+1}^{2} & 0 & 0\\ 0 & 0 & \ddots & 0\\ 0 & 0 & 0 & H_{k+1}^{n} \end{array}\right]\;\left[\begin{array}{c} \partial {\text{x}}_{k+1}^{1}\\ \partial {\text{x}}_{k+1}^{2}\\ \vdots \\ \partial {\text{x}}_{k+1}^{n} \end{array}\right]+\left[\begin{array}{c} {\text{η}}_{k+1}^{1}\\ {\text{η}}_{k+1}^{2}\\ \vdots \\ {\text{η}}_{k+1}^{n} \end{array}\right]$$

where $\Phi_{k,k+1}^{n}$ is the *n*^th^ block filter transition matrix, $\partial {\text{x}}_{k+1}^{n}$ is the *n*^th^ block filter states (21 state model), $\partial {\text{z}}_{k+1}^{n}$ is the misclosure vector from the *n*^th^ block filter of the observations, ${\text{w}}_{k}^{n}$ is the process driving noise of the *n*^th^ block filter, and ${\text{η}}_{k+1}^{n}$ is the measurement noise of the *n*^th^ block filter.

The stacked transition matrix of (7) and the design matrix of (8) are block diagonal. This important characteristic makes the block filters operate independently, unless additional updates are applied. Thus, if the stacked filter operated without additional updates, the block results would theoretically be identical to independent INS filters. In practice however, round off errors and small computational correlations between block filters result in small differences (*i.e.*, the position varies by a few centimetres).

During a GPS update, each block filter requires its own misclosure vector, derived from the GPS observations. However, if each block requires its own misclosure vector, the GPS observations must be repeatedly used for each IMU, thereby directly violating fundamental Kalman filter theory [35]. The stacked filter innovation vector would have the form:

(9) $$\left[\begin{array}{c} {\text{υ}}_{k}^{1}\\ {\text{υ}}_{k}^{2}\\ \vdots \\ {\text{υ}}_{k}^{n} \end{array}\right]=\left[\begin{array}{c} {\tilde{\text{P}}}_{k}\\ {\tilde{\text{P}}}_{k}\\ \vdots \\ {\tilde{\text{P}}}_{k} \end{array}\right]-\left[\begin{array}{c} h\left({\text{x}}_{\;k}^{(-)1}\right)\\ h\left({\text{x}}_{\;k}^{(-)2}\right)\\ \vdots \\ h\left({\text{x}}_{\;k}^{(-)n}\right) \end{array}\right]$$

where **P̃***_k_* is the GPS observation vector of output by the receiver and $h\left({\text{x}}_{\;k}^{(-)n}\right)$ is the predicted observation vector derived from the observation equation using the n^th^ block state vector of the k^th^ epoch.

### 3.1. Stacked Filter Relative Updates

Because the stacked filter contains multiple position, velocity and attitude states, one for each IMU, the filter can be updated with relative position, velocity and attitude (PVA) information that is known *a priori*. A relative update does not constrain the absolute value of the parameters within the block filters, but constrains relative PVA between the IMUs. It also aids in the estimation of the bias and scale factors of the IMUs.

The inter-IMU vector is measured in one of the IMU’s body frame and is computed by differencing the lever arms (*i.e.*, the vector from the GPS antenna to the IMU in the body frame). The relative position observation equation is given by:

(10) $${\text{L}}^{2,1}={\hat{\text{r}}}^{1}-{\hat{\text{r}}}^{2}$$

where **r̂**^1^ is the estimated position vector of the 1^st^ block filter and **L**^2,1^ is the *a priori* known vector between the IMUs.

It is important to note that by differencing the lever arms to generate the inter-IMU vector, the lever arms must be in the same frame and not their respective body frames. Since the Earth Centered Earth Fixed (ECEF) frame was used as the navigation frame, the inter-IMU vector must be rotated into that frame. Consequently, there is an inherent relationship between the efficacy of the relative position update (RUPT) and the error in the orientation of the body frame relative to the ECEF frame.

The update is applied periodically to facilitate a convergence of the block INS filter, reduces numerical computations and limits the inter-block correlation accumulation. Using experimental filter tuning, a periodicity of 6 s and a standard deviation of 1 cm (a diagonal matrix) provided the best performance.

The relative velocity of a point on a moving rigid body is given by Marion & Thornton [36]. In the context of two rigidly mounted IMUs, the relative velocity is expressed as:

(11) $${\dot{\text{L}}}^{2,1}={\text{ω}}_{1}\times {\text{L}}^{2,1}$$

where **L̇**^2,1^ is the relative velocity between the IMUs 2 and 1, **ω**_1_ is the angular velocity vector measured by IMU 1, and **L**^2,1^ is the vector between IMUs 1 and 2.

The vector between the IMUs is assumed to be known *a priori* and the angular velocity vector is observed by the first inertial unit. This update therefore derives its input from the observation of the IMU. The accuracy is a function of the noise characteristics of the IMU and the filter’s ability to correctly estimate the systematic IMU errors. The relative velocity observation equation is given by:

(12) $${\dot{\text{L}}}^{2,1}={\text{ω}}_{1}\times {\text{L}}^{2,1}$$

where **v̂**^1^ is the velocity vector of the 1^st^ block filter and **v̂**^2^ is the velocity vector of the 2^nd^ block filter.

As with the relative position update, the relative velocity observation is derived in the body frame and must be rotated into the navigation frame, thus creating a similar relationship between the error of the rotation and the RVUPT. The standard deviation used for RVUPTs was 2 cm/s and was derived using the propagation of variances of Equation (11), assuming nominal values of the IMUs noise characteristics and the accuracy of the known lever arm.

The relative attitude update follows a similar procedure to the relative position update. The misclosure vector is formed using the difference in estimated Euler angles of each IMU and the pre-surveyed Euler angles describing the rotation between them. In this research the IMUs are fixed on the same platform and mounted on adjacent faces thereby allowing simple Euler angle identification. The relative attitude observation equation is given by:

(13) $$\begin{array}{l} \theta_{B1}^{B2}={\hat{\theta }}^{B1}-{\hat{\theta }}^{B2}\\ \phi_{B1}^{B2}={\hat{\phi }}^{B1}-{\hat{\phi }}^{B2}\\ \psi_{B1}^{B2}={\hat{\psi }}^{B1}-{\hat{\psi }}^{B2} \end{array}$$

where $\theta_{B1}^{B2}$ is the roll between the first and second IMU body frames, $\phi_{B1}^{B2}$ is the pitch between the first and second IMU body frames, $\psi_{B1}^{B2}$ is the yaw between the first and second IMU body frames and *θ^B^*^1^, *φ^B^*^1^, *ψ^B^*^1^ is the roll, pitch and yaw of the first IMU, respectively. The standard deviation of this observation is 0.1 rad (*i.e.*, 5.7°).

### 3.2. Stacked Filter Fault Detection and Exclusion of GPS Measurements

Since GPS observations are repeated within the stacked filter, the FDE process is slightly modified for GPS observations. The modification eliminates the possibility that GPS observations may be rejected for one block filter and accepted for another, while at the same time improving the reliability of the fault detection scheme. The effect of the blunder vector and its mapping matrix on the observation vector can be described as:

(14) $${\text{l}}_{\text{k}}={\text{H}}_{\text{k}}{\text{x}}_{\text{k}}+{\text{M}}_{\text{k}}\nabla_{\text{k}}+{\text{ɛ}}_{\text{k}}$$

where M_k_ is the blunder mapping matrix and ∇_k_ the vector of known blunders.

It is in this equation that the FDE algorithm will be modified to test a series of observations (corresponding to a single GPS measurement) rather than elements of the innovation sequence. The M matrix is generated based on the GPS observations and number of IMUs used. For example, the M matrix with three pseudoranges, repeated for two block filters in a stacked filter, with a single fault in the first observation will be *M* = [1 0 0 1 0 0 ]^T^ . The test statistic is then computed from with direct reference to the GPS observations as [37]:

(15) $${\text{T}}_{\text{k}}={\text{υ}}_{\text{k}}^{\text{T}}{\text{C}}_{{\text{υ}}_{\text{k}}}^{-1}{\text{M}}_{\text{k}}{\left({\text{M}}_{\text{k}}^{\text{T}}{\text{C}}_{{\text{υ}}_{\text{k}}}^{-1}{\text{M}}_{\text{k}}\right)}^{-1}{\text{M}}_{\text{k}}^{\text{T}}{\text{C}}_{{\text{υ}}_{\text{k}}}^{-1}{\text{υ}}_{\text{k}}$$

The test statistic is a chi-squared distribution. The null and alternate hypotheses are:

(16) $${{\text{T}}_{\text{k}}|}_{{\text{H}}_{0}}∼\chi^{2}\left(\text{d},0\right)$$

(17) $${{\text{T}}_{\text{k}}|}_{{\text{H}}_{\text{a}}}∼\chi^{2}(\text{d},\delta_{0}^{2}),$$

where d is the degree of freedom (the number of times an observation is used) and δ_0_ is the non-centrality parameter. With these hypotheses, the test is conducted by rejecting the null hypothesis if ${\text{T}}_{\text{k}}\geq \chi_{\alpha }^{2}(\text{d},0)$.

The MDB of the stacked filter can then be determined as:

(18) $$MDB_{\text{Stacked Filter}}=\frac{\delta_{0}}{{\left(M^{T}C_{\text{υ}}^{-1}M\right)}^{1/2}}$$

Assuming that the innovation covariance matrix is equivalent between block filters, the improvement in the MDB *versus* a SINS MDB is 1/√n.

## 4. Federated Filter Fusion

To the authors’ knowledge, there has been no published work in the domain of decentralized filters incorporating multiple IMUs. Federated filters were introduced in the late 1980s and early 90s for GPS and INS integration (e.g., [38,39]), but have not been extended to the multi-IMU case. Federated filters utilizing several other navigation systems such as radar altimeters, terrain aided navigation systems and synthetic aperture radar have been discussed, but not restricted to IMUs [40,41].

Federated filtering is defined herein as a decentralized filter that incorporates information sharing between local and master filters. References [41] and [42] show that the information conservation principle within the federated filter is optimally equal to the centralized version, although practically this may not always be the case. A rigorous derivation is available in [38]. The method of sharing information varies, depending on the type of local and master filters, but there are typically four genres of sharing information: no reset, fusion reset, zero reset and cascaded. Federated No Reset (FNR) and Federated Fusion Reset (FFR) are used herein as the other methods of sharing information are not conducive to inertial navigation systems.

The federated filters discussed herein contain common states. Specifically, the shared states are position (**r**), velocity (**v**) and the Euler angles representing the rotation from the body frame to the ECEF frame (**α**).The local filters estimate these parameters as part of their 21-state filters. The master fusion filter (or least squares estimator as the case may be) also contains the same shared states (**r**, **v** and **α**). In this manner, only these states are shared, all biases and scale factors within the local filters remain unmodified. It is important to note that the Euler angles of each IMU are rotated into a virtual IMU frame and this rotation is assumed to be known *a priori*.

**Figure 3.**
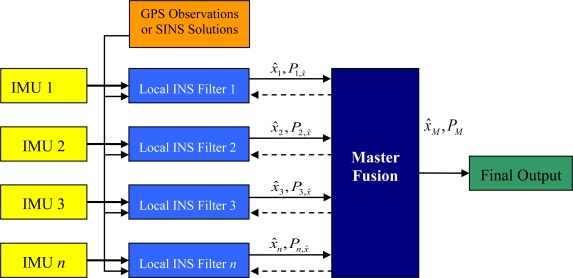
Federated Filter Architecture of Multiple IMUs.

The reference data of the local filters can be formed by one of two methods. The first method is to use GPS observations, whereby each local filter operates in a tightly coupled manner (*i.e.*, GPS observations are used in each of the local filters). The second method is to use one of the IMUs to form an INS aided by the GPS observations, the output thereof providing updates to the local filters. In this manner, the federated filter operates in a loosely coupled architecture. If the INS provides the reference to the local filter, it also provides a time correlated input into the observations of the local filters. This time correlation violates the rules of observation input into a filter and therefore would generate an overly optimistic variance of the states. The federated filter architecture for multiple IMUs is shown in Figure 3. The dashed line represents the sharing information algorithm.

### 4.1. Federated No Reset Filter

The FNR filter is fundamentally equivalent to running each IMU through an INS filter and combining the final results of each solution via least squares. The master fusion is performed via least squares with each local filter’s PVA providing the observations.

Thus, if there are five IMUs, the master estimator contains 45 observations and correspondingly, a 45 × 45 observation covariance matrix. The master’s input observation covariance matrix is block diagonal, however the internal PVA correlation remains within the off diagonal elements (*i.e*., [*P_n_*]_9_*_x_*_9_ is not diagonal). The PVA of the local filters is in reality correlated as a result of using the same GPS observations and moreover by potentially similar dynamics if the IMUs are rigidly mounted together. Therefore the input observation covariance matrix is scaled by n^−1^ to reduce the weight of each correlated observation.

### 4.2. Federated Fusion Reset Filter

The FFR filter has a similar structure to the FNR filter, but the master filter parameters (and its corresponding covariance matrix) are shared with the local filters. The information factor for each local INS filter is n^−1^ because the IMUs are all the same brand and model. The input to the master fusion is the same as the FNR filter. Furthermore, since the states of the INS extended Kalman filter are zero, the PVA of the master fusion replaces the PVA used to provide the expansion point, rather than the actual values in the state vector. The covariance information of the local filters, however, is replaced with the actual values from the local and master filters. Additionally, because correlation develops within the local filter PVA states and IMU error states, these intra filter correlations must be set to zero, otherwise the filter will diverge. Further, the covariance replacement of the i^th^ local filter with the master state covariance matrix is as follows, the first nine states representing the PVA having been replaced:

(19) $$P_{i}=\left[\begin{array}{cc} \frac{1}{n}(P_{M_{9x9}}) & 0_{9x12}\\ 0_{9x12} & P_{i_{12x12}} \end{array}\right]$$

where *P_i_* represents the covariance matrix of the i*^th^* local filter and *P_M_* represents the covariance matrix of the master filter. *P*_*i*_12*x*12__ remains unmodified during the covariance replacement because it contains the bias and scale factors of the i*^th^* IMU which are not shared between the local and master filters.

### 4.3. Comparison of Architectures

Table 1 shows a comparison of the different architectures described in the chapter and each architecture’s strengths and weaknesses.

### 4.4. Filter Tuning

Tuning the filters presented a significant (and time consuming) problem. There are five tunable parameters for each sensor (*i.e.*, axis) within an IMU. With a five-IMU configuration there are potentially over 120 potential parameters to tune, aside from parameters customized for each architecture (e.g., federated filter sharing information rate). It should be noted that in the VIMU case, only one IMU (*i.e.*, the VIMU) requires tuning. For the stacked and federated filters, achieving a high level of tuning for each parameter is simply unrealistic given the quantity. It is conceded that there could be better results with more customized filter tuning for each architecture type. However, the results are more representative to those available in an industrial environment where each sensor could not be individually tuned.

Therefore, a generic set of tuning parameters was used for each data set for all IMUs. Only minor modifications to the spectral densities were allowed to accommodate each sensor noise range. Consequently, the same parameters used in the single IMU solution were used in every other multi-IMU solution. Although the solutions may be somewhat sub-optimal, the methodology facilitates better filter performance comparisons, rather than tuning performance comparisons.

## 5. Data Collection Environments

**Figure 4.**
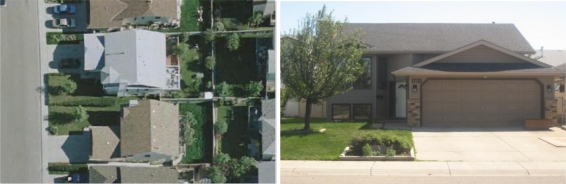
Residential House used for Data Collection.

Data was collected in two environments: a typical North American residential home and inside the Olympic Oval of the University of Calgary. The residential home, as shown in Figure 4, provided an excellent example of an area where GPS is attenuated by 4 to 18 dB and delivers standalone horizontal accuracies of several metres. Although GPS could provide reasonable accuracy in such an environment, the benefit of an integrated system to reject multipath is valuable and the ability to position an individual in a specific room of the home can be of great value to first responders.

**Figure 5.**
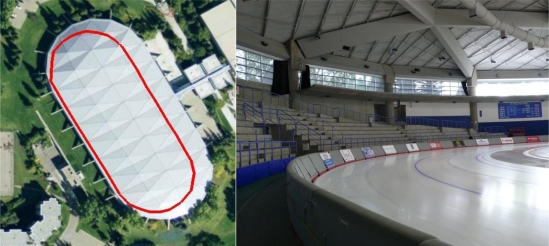
Olympic Oval (Left: roof top with trajectory in red, Right: inside showing track and ice level).

The Olympic Oval, shown in Figure 5, is an ideal location for indoor testing as GPS signals are attenuated by 25 to 35 dB, but yet can be tracked with high sensitivity receivers. The oval running track is 450 m long. Because of the severe signal attenuation and the building characteristics, the effects of multipath and noise are large, often to a point where the GPS solution is completely unreliable and unusable. In this environment there must be an integrated system to provide useful navigation information.

### 5.1. Data Collection Set Up

To collect the data, the test subject carried a rigid aluminum backpack to house a tactical grade reference INS, two laptops to collect the GPS and IMU data, and batteries to power the equipment. A NovAtel SPAN system was used to provide the reference solution. It consists of a Honeywell HG1700 AG58 IMU and a NovAtel OEM4 GPS receiver. The data in this case was differentially post-processed with a nearby (<1 km) reference station to provide a reference trajectory. The data was processed with NovAtel’s Inertial Explorer software in forward and reverse directions, smoothed using RTS smoothing [43] and then combined for the final reference solution. The reference solution was accurate to within a few metres in the Oval, and better than 0.5 m in the residential house.

**Figure 6.**
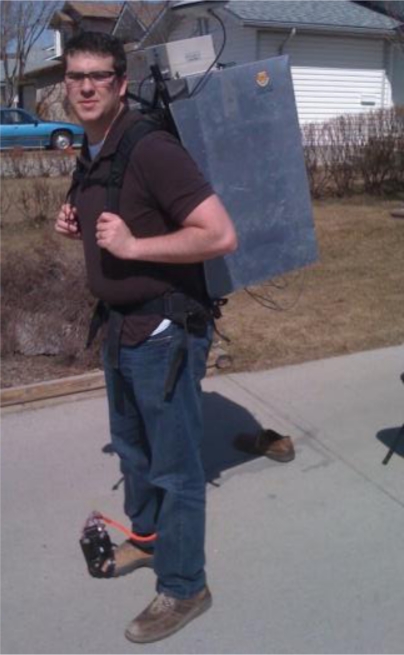
Rigidly Mounted IMUs on the Foot.

The high sensitivity GPS receiver used was a u-blox Antaris 4 Precision Timing AEK-4T evaluation kit with firmware 5.0. The antenna was a u-blox ANN-MS, designed and manufactured by Allis Communications Co Ltd as antenna M827B [44]. The antenna was attached to the top of the backpack, rather than the head, to avoid the effects of antenna detuning [45]. All GPS data was differentially processed to eliminate satellite position and clock errors and reduce the effect of atmospheric errors. This enabled a clear analysis of the multi IMU method rather than errors derived from single point (GPS) positioning. The IMUs used were Cloudcap Technology’s Crista IMUs. The error characteristics of the Crista IMU and the HG1700 AG11-58 tactical grade are shown in Table 2. Figure 6 shows a picture of the IMUs rigidly mounted on a platform attached to the author’s foot.

Although the lever arm is time variant, the variation is symmetric about the fixed lever arm. It is under this assumption that solutions can be compared to within a decimetre error envelope.

## 6. Residential House Data Set

### 6.1. VIMU Results

**Figure 7.**
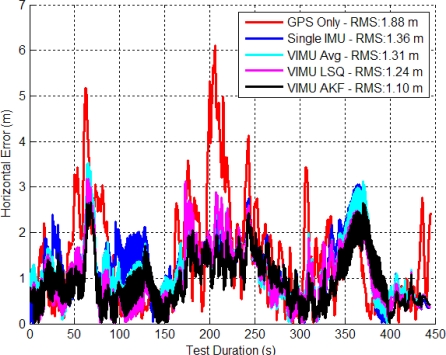
VIMU Horizontal Errors (Five IMUs Used in Residential Data Set).

Figure 7 shows the time series’ horizontal errors for the three VIMU fusion methods, and the standalone GPS and typical Single INS (SINS) for comparison. The horizontal error RMS values are shown in the legend and indicate that moving to the adaptive filter provides a 10.1% and 6.6% improvement in accuracy than averaging and the Least-Squares (LSQ) methods, respectively. At time 100 s in Figure 7, the user encounters open sky and the Adaptive Kalman Filter (AKF) quickly accepts the GPS observations, whereas the VIMU and SINS solutions take nearly 35 s longer to converge. When in the house basement where standalone GPS has a six metre horizontal error, the VIMU filters maintain a two metre accuracy whereas the SINS solutions achieve only a three to four metre accuracy.

The VIMU solutions contain more noise as a result of the decreased spectral densities used within the filter. This effect was amplified when GPS measurements were stronger (*i.e.*, signal power increased) and the filter weighed the observations more heavily, thus shifting the position. As the filter de-weighed the GPS measurements as signal power decreased, the navigation solution displayed a smoother trajectory.

**Figure 8.**
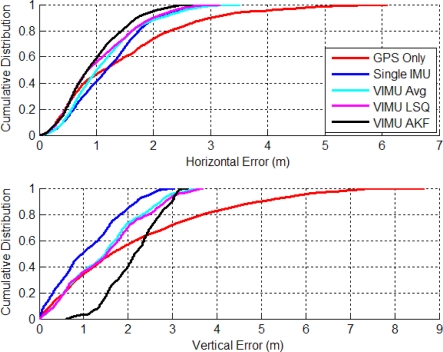
CD of Horizontal and Vertical Errors (Residential Data Set).

The cumulative densities (CDs) of the horizontal and vertical errors are shown in Figure 8. The VIMU AKF performance was best in the horizontal plane and poorest in the vertical axis. In the latter, the VIMUs behaved similarly to the SINS solution, although it was clear that there was no improvement with the VIMU average and VIMU LSQ solutions.

### 6.2. Stacked and Federated Filter Accuracy

**Figure 9.**
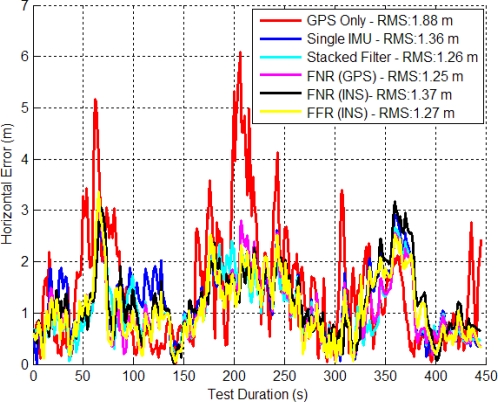
Stacked and Federated Filter Horizontal Errors (Residential Data Set).

The stacked filter, FNR and FFR filter’s horizontal errors are shown in Figure 9. The FNR (GPS) filter (FNR (GPS) refers to the federated filter with GPS observations as the reference) provided the best solution between the stacked and federated filters but only by less than one percent.

Since the GPS signal strength is still reasonable in this environment, the additional information contained within the relative updates did not further improve the accuracy of the final solution. This indicates that the filter’s biases and scale factors had been resolved and other unmodeled error sources begin to dominate the solution’s accuracy. The FNR (INS) performed 6.3% worse than the FNR (GPS), which indicates that using the raw ranges of the GPS receiver as input to each local filter is superior.

**Figure 10.**
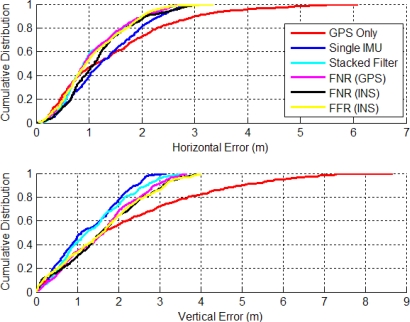
CD of Horizontal and Vertical Errors for Stacked and Federated (Residential Data Set).

Figure 10 shows the CDs of the horizontal and vertical errors. The horizontal distributions have a slightly improved performance with more results better than 1 m. For example, the SINS filter solution is better than 1 m 38.5% of the time, whereas the corresponding stacked filter value is 58.3% and that for the FNR (GPS) filter is 56.5%. In the vertical channel the stacked filter had the best CD with 41.5% of errors less than 1m compared to the FNR (GPS) at 33.9% less than 1 m error.

### 6.3. Filters Position Accuracy vs. Number of IMUs

**Figure 11.**
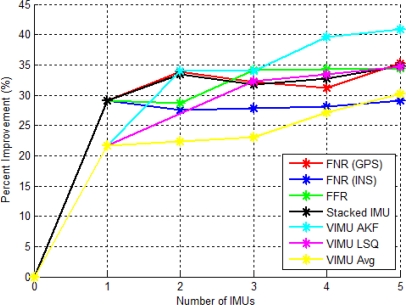
VIMU Accuracy as a Function of IMUs Used (Residential Data Set).

Figure 11 shows the RMS percent improvement relative to that of a standalone GPS solution as a function of IMUs. The AKF method had the largest increase when a second IMU was added, although this dramatic increase was not sustained with the addition of the third, fourth and fifth IMU. This is a direct result of estimating the angular acceleration within its filter. Interestingly, applying the averaging technique with five IMUs was less accurate than with two IMUs using the LSQ or AKF method. This confirms that estimating the angular acceleration had a positive impact on the accuracy of the navigation solution, even more so than the number of IMUs used. This was an important practical finding, which makes the use of a dual inertial system considerably more attractive.

The stacked filter showed the largest percent increase with two IMUs, but then decreased with the addition of the third and fourth IMU. The third and fourth IMUs were among the least accurate SINS solutions. Thus, when the filter combined the block filter solutions, the final solution was degraded. This contradicts the hypothesis that the relative updates would have provided additional information to improve the accuracy of each block filter. This contradiction is refuted with the data set from the Olympic Oval, which shows that in the absence of reasonable GPS observability, the relative updates significantly improve the navigation solution.

The FNR (GPS) results followed a similar trend to that of the block filter, again suggesting that the relative updates were providing little improvement to navigation solutions in this case. The FFR (INS) filter performance plateaued at the third IMU and had similar results with three to five IMUs, only increasing 0.1% per additional IMU. The FNR (INS) percentage improvement was minute with only 0.3, 0.4 and 1.2% for each additional IMU.

Consistent with the results of the VIMU architecture in Section 6.1, the addition of the second IMU had the largest percentage increase, even more so than the third, fourth or fifth IMU. This suggests that if two IMUs are used, the stacked, FNR (GPS) or VIMU AKF all show similar performance. However, when using more than two IMUs, the solution accuracy improves at a lower rate.

## 7. Olympic Oval Data Set

**Figure 12.**
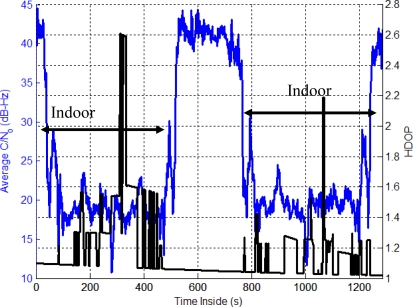
Average C/N_o_ and HDOP (Olympic Oval Data Set).

The Olympic Oval presents a different approach to that of Section 6 as in this environment, GPS will not provide acceptable performance for most applications and an integrated system is needed. Figure 12 shows an average power drop of 24 dB inside the Oval while the HDOP occasionally doubles. In addition, due to the material used during construction and the geometry of the building, multipath is high. This figure also shows the relative power increases when the user is located outside to allow the reference solution to re-estimate the IMU errors (*i.e.*, 500 to 750 s).

### 7.1. VIMU Results

**Figure 13.**
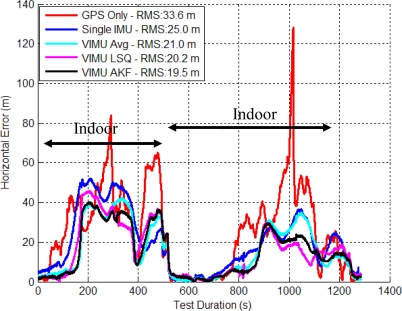
VIMU Horizontal Errors (Five IMUs Used in Olympic Oval Data Set).

The VIMU horizontal errors are shown in Figure 13. The horizontal error improvement is more significant than that of the residential data set (e.g., Section 6.1). The VIMU average provided a 37.7% improvement, and the LSQ and AKF methods were similar with 40.1% and 42.1% improvements, respectively. Further investigation showed that the results are also hindered by time tagging issues due to several unsynchronized IMUs.

The VIMU tends to diverge much more slowly when entering the indoors and converges much more quickly when exiting, compared to the SINS solution. That said, at time 185 s, the solution very quickly diverged from a 6 m error to nearly a 40 m error. This was a direct result of a strong multipath signal that had a high C/N_o_. The filter consequently overweighed the pseudorange and the VIMU filters were unable to reject this information. This effect has been seen in all the filters during this research and presents a problem that could not be solved without manual intervention of the observation covariance matrix.

**Figure 14.**
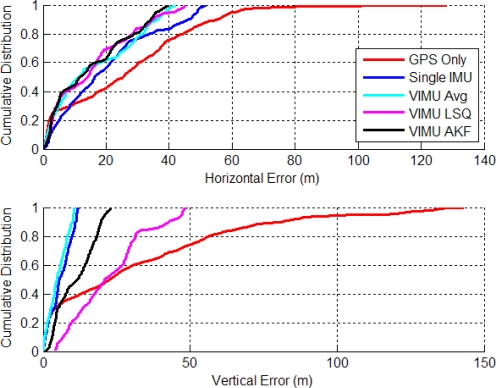
CD of VIMU Horizontal and Vertical Errors (Five IMUs Used in Olympic Oval Data Set).

Figure 14 shows the CD of the horizontal and vertical errors. The VIMU’s horizontal errors showed superior performance at a 40% error. This revealed a distinct advantage over the SINS solutions. However, beyond 40% the advantage was less pronounced and provided only marginal improvement compared to the SINS solution. In the vertical axis the LSQ and the AKF drifted but then slowly converged when GPS was less attenuated. This convergence was much slower than in the SINS and VIMU average solutions.

### 7.2. Stacked and Federated Filter Results

**Figure 15.**
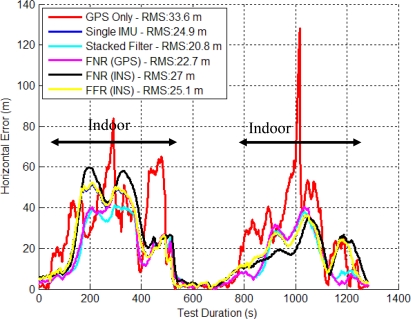
Horizontal Error of Stacked and Federated Filters (Five IMUs Used in Olympic Oval Data Set).

Figure 15 provides the stacked and federated filter horizontal error results. The best solution was the stacked filter which outperformed its FNR (GPS) counterpart by 8.9%. This is evidence of the effectiveness of the relative updates providing more information to the filter assisting in constraining the divergence of the system when GPS is providing poor observations. The FNR (GPS) filter again provided more accurate results to the FNR (INS) and FFR (INS), which provided similar results as the SINS solutions.

The SINS and the FFR (INS) error profiles in Figure 15 show a similar result. This occurred because the reference INS in the FFR was the same single INS plotted in Figure 15. This introduces a concept where the reference local filter was aiding the other local filters to follow its trajectory because the input “observations” were time correlated. This, in some cases, is to the detriment of a federated filter using one local filter as its reference solution for other local filters. This result confirmed that the reference system data must yield to assumptions of the Kalman filter namely that there is no time correlation of the measurement errors [35].

**Figure 16.**
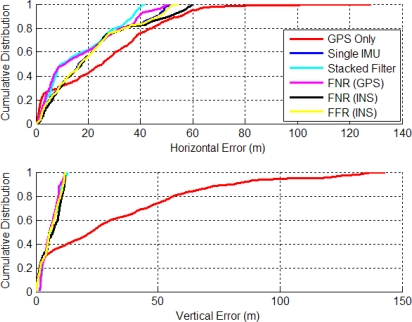
CD of Horizontal and Vertical Errors for Stacked and Federated Filters (Olympic Oval Data Set).

Figure 16 shows the CD of the horizontal and vertical errors. The stacked filter provided a reasonable improvement at 90% CD where it outperformed the FNR (GPS), but followed a similar trend at lower percentages. Both the FNR (GPS) and stacked filter behaved similarly below 80%, which showed that, in terms of the distribution, the relative updates were providing improvement at times when the FNR (GPS) did not.

**Figure 17.**
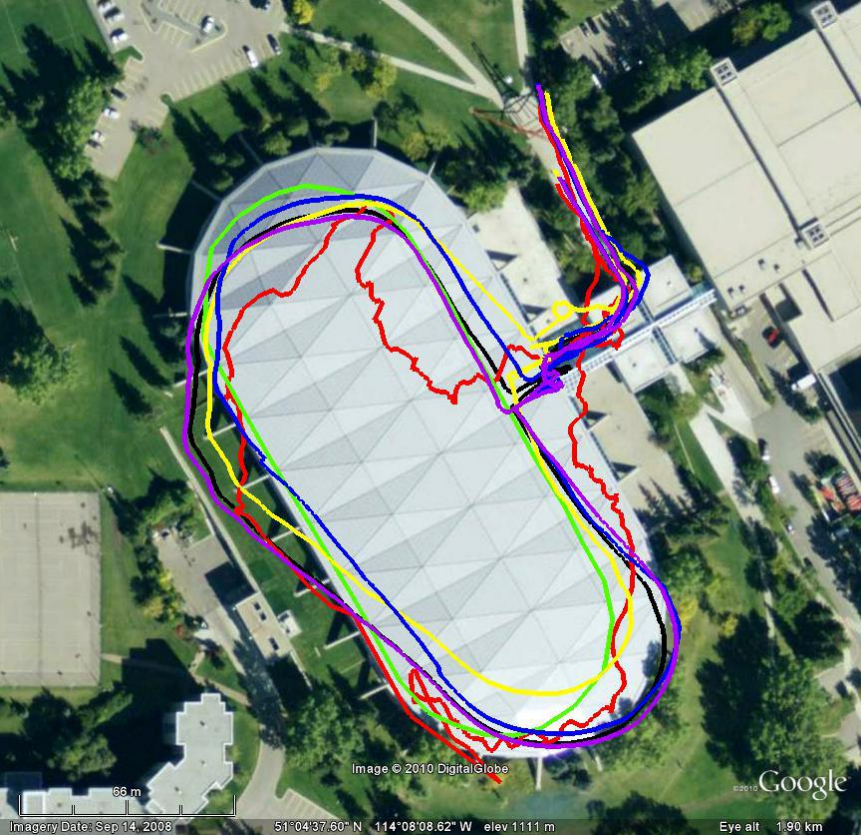
Loop 2 (Clock Wise) Map View of Best Performing Filters—Truth Solution ( 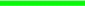), Standalone GPS Solution ( 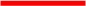), SINS ( 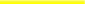), VIMU (AKF) ( 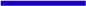), Stacked Filter (—), FNR (GPS) ( 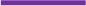).

To compare the results of each filter, Figure 17 shows a map with the trajectories of each architecture best solution (*i.e.*, least amount of RMS error). A standalone GPS solution and a SINS solution are also provided for context. During the test the subject walked around the Oval in a clockwise direction. In this trajectory, the SINS was originally correctly providing a good heading, but had acquired an along-track error that provided the large horizontal error shown. By the time the user exited the track, the SINS solution contained the largest heading error. This was indicative of the heading degrading during the time indoor, which was less prominent in the multi-IMU architectures. With remarkable accuracy, the FNR (GPS) and the stacked filter had aligned themselves with the truth trajectory at the north east corner and appear to have a very accurate heading, better than 10 degrees after nearly 350 s.

For the Oval data, the user entered and exited the track at the same point and therefore provided an interesting metric to compare the solutions. The FNR (GPS) filter only deviated by 2.5 m, the SINS difference was 13.5 m and the standalone GPS solution had a 49.3 m difference. The same check of the reference system yielded a 5.1 m difference.

### 7.3. Position Accuracy versus Number of IMUs

**Figure 18.**
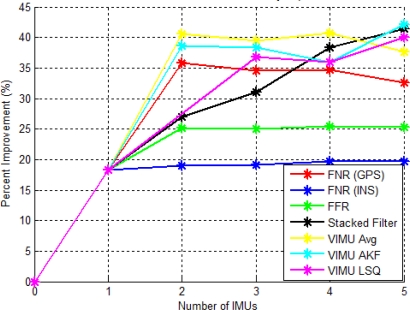
VIMU Accuracy Improvement as a Function of IMUs Used (Olympic Oval Data Set).

The accuracy of each architecture as a function of the number of IMUs is shown in Figure 18. This figure provides an indication of the weakness of the VIMU time tagging as discussed in [3]. Because of this issue, the incremental improvement for the VIMU fusion methods was modest. In this case the VIMU AKF provided the best solution, despite marginal time synchronization issues.

The stacked filter had a linear improvement for each additional IMU of about 3 to 7% per IMU added. This again indicates the value of the relative updates, as each additional IMU provided additional relative information to improve the accuracy of the solution and the error states within the block filters. The FNR (INS) and the FFR (INS) results did not increase linearly, but plateaued similarly to the results of two IMUs. The FNR (GPS) slightly decreased with each additional IMU in excess of two.

The FNR (INS) and FFR (INS) results were very similar to the residential data set with very moderate improvements as each IMU was added. The FNR (GPS) also had similar results between data sets with a slight decrease in performance with more IMUs. The two data sets confirm that the federated filter architecture did not increase the accuracy, but merely processed the data in a similar manner to that of the centralized version.

## 8. Processing Speed of Architectures and Number of IMUs

**Figure 19.**
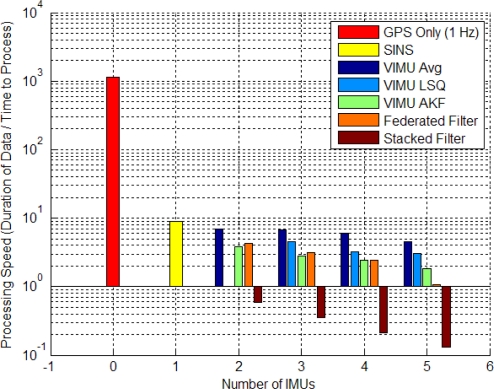
Processing Speed of Various Architectures.

There is a large difference in the computer processing speed of each architecture and for the number of IMUs used. An exact comparison of the computational load is beyond the scope of this paper, but Figure 19 shows the processing rate of each architecture and the number of IMUs added for the software developed by the author.

All data was processed on an Intel Core 2 Quad CPU with 3.25 GB of RAM. This analysis is merely intended to be comparative, since there are numerous factors that determine processing speed. The slowest architecture was the stacked filter. This was mostly due to the inversion required for the gain matrix computation, which has n times m rows and columns (n is the number of IMUs and m is the number of GPS observations); propagating the filter forward was also a burden. This was the only filter that could not run in real time. For those interested in operating a stacked filter in real time, several processing enhancements could be made to reduce the computational load. These include processing observations sequentially, using integer based data types, reducing the IMU data rates, propagating the filter for longer intervals rather than shorter more frequent ones or using factorization methods such as Cholesky decomposition. Readers are referred to [46] for other optimization techniques. The VIMU AKF was able to process faster than the federated filters, an interesting note considering the VIMU AKF produced solutions at 100 Hz whereas the federated filters operate at 20 Hz. These results are largely influenced by I/O processes such as the input and output of the filters data, which include PVA navigation parameters and estimated variances, biases and scale factors for each IMU with their respective variances, MDB information, satellite number and DOP information. Thus, in the event of a five IMU federated filter, the output was five times greater than that of a SINS filter.

## 9. Conclusions

Three architectures were proposed for which multi-IMU data can be fused to provide improved navigation performance. The filters proposed specifically assess the integration schemes within the scope of pedestrian navigation. The objective was to compare the results of three architectures and provide insight into the advantages and disadvantages of each, providing a better understanding of the accuracy and availability for each filter.

The stacked filter provided better results compared to its federated reset free counterpart, which showcases the use of relative updates and a better fault detection algorithm. Although the improvement was minor in the residential data set, the filter was already operating at a high performance level with the use of only moderately attenuated GPS signals. In the Olympic Oval data set, the stacked filter performed 9% better with five IMUs than the federated reset free filter. The multi-IMU federated filters accuracy reached a maximum with two IMUs, whereas the stacked filter accuracy linearly increases 3 to 7% with each additional IMU. This suggests that the relative updates provide a linear relationship with the number of IMUs, at least up to five units.

When GPS measurements were used as the reference information for the local filters of the federated filter, the performance was 15% better than when a single INS solution was used as the reference for the federated filter. The time correlation of the output of the INS solution resulted in a dramatic decrease in performance of the local filters.

Within the VIMU scope, FDE is not practical unless the systematic errors have been removed prior to testing for faults. Performance within the FDE is severely hindered by the dynamics of the IMU and the magnitude of the scale factors, biases and acceleration based gyroscope errors. There is also no evidence within this research to suggest that FDE on IMU measurements would increase navigation accuracy or availability; the primary interests of pedestrian navigation.

Processing times of the filters differ, but the stacked filter requires the most processing time, followed by the federated filters, VIMU AKF, VIMU LSQ and VIMU average.

## Figures and Tables

**Table 1.** Comparison of the Various Architectures.

**Filter/Estimation Characteristic**	**VIMU**	**Centralized**	**Federated Filter**
Enhanced GPS Observation FDE	No	Yes	No
IMU Observation FDE	Not Recommended	No	No
Reduced Noise at Mechanization Input	Yes	No	No
Constrains Estimator using Relative PVA	No	Yes	No
Estimates Each IMUs Bias and Scale Factor	No	Yes	Yes
IMU Time Synchronization Not Required	No	Yes	Yes

**Table 2.** Reference and MEMS Grade IMU Maximum Errors.

		**HG1700 AG11-58 Tactical Grade IMU**	**Cloudcap Crista MEMS Grade IMU**

Accelerometer	In Run Bias (mg)	1	51
	Turn on Bias (mg)	-	30
	Scale Factor (PPM)	300	10,000
	Random Walk (g/√Hz)	2.16 × 10^−6^	370 × 10^−6^

Gyro	In Run Bias (°/h)	1	2,160
	Turn on Bias (°/h)	-	5,400
	Scale Factor (PPM)	150	10,000
	Random Walk (°/h/√Hz)	.5	226.8
